# Geriatric Nutritional Risk Index as a Screening Tool to Identify Patients with Malnutrition at a High Risk of In-Hospital Mortality among Elderly Patients with Femoral Fractures—A Retrospective Study in a Level I Trauma Center

**DOI:** 10.3390/ijerph17238920

**Published:** 2020-11-30

**Authors:** Wei-Ti Su, Shao-Chun Wu, Chun-Ying Huang, Sheng-En Chou, Ching-Hua Tsai, Chi Li, Shiun-Yuan Hsu, Ching-Hua Hsieh

**Affiliations:** 1 Department of Trauma Surgery, Kaohsiung Chang Gung Memorial Hospital, Chang Gung University College of Medicine, Kaohsiung 803, Taiwan; s101132@adm.cgmh.org.tw (W.-T.S.); junyinhaung@yahoo.com.tw (C.-Y.H.); athenechou@gmail.com (S.-E.C.); tsai1737@cloud.cgmh.org.tw (C.-H.T.); foocollie7@gmail.com (C.L.); ah.lucy@hotmail.com (S.-Y.H.); 2 Department of Anesthesiology, Kaohsiung Chang Gung Memorial Hospital, Chang Gung University College of Medicine, Kaohsiung 803, Taiwan; shaochunwu@gmail.com; 3 Department of Plastic Surgery, Kaohsiung Chang Gung Memorial Hospital, Chang Gung University College of Medicine, Kaohsiung 803, Taiwan

**Keywords:** elderly, femoral bone fracture, malnutrition, mortality, geriatric nutritional risk index, trauma

## Abstract

Background: Malnutrition is frequently underdiagnosed in geriatric patients and is considered to be a contributing factor for worse outcomes during hospitalization. In addition, elderly patients who undergo trauma are often malnourished at the time of incurring fractures. The geriatric nutritional risk index (GNRI), calculated based on the serum albumin level and the ratio of present body weight to ideal body weight, was proposed for the assessment of the nutritional status of elderly patients with various illnesses. This study aimed to investigate whether the GNRI has a prognostic value that links the nutritional status and mortality outcomes of elderly patients who have previously undergone trauma with femoral fractures. Methods: From January 1, 2009 to December 31, 2019, a total of 678 elderly patients with femoral fractures were categorized into four nutritional risk groups: a major-risk group (GNRI < 82; group 1, *n* = 127), moderate-risk group (GNRI 82–92; group 2, *n* = 179), low-risk group (GNRI 92–98; group 3, *n* = 123), and no-risk group (GNRI > 98; group 4, *n* = 249). To minimize the confounding effects of sex, age, preexisting comorbidities, and injury severity of patients on outcome measurements, propensity score-matched patient cohorts were created to assess the impact of patients being in different nutritional risk groups on the in-hospital mortality outcomes against the no-risk group. Results: The patients in groups 1–3 were significantly older and presented a significantly lower body mass index and lower serum albumin levels than those in group 4. Compared with patients in group 4 (3.6%), a significantly higher mortality rate was found in the patients in group 1 (17.3%, *p* < 0.001), but not in those in group 2 (6.7%) or group 3 (2.4%). The study of propensity score-matched patient cohorts provided similar results; group 1 patients had significantly higher odds of mortality than group 4 patients (odds ratio, 6.3; 95% confidence interval, 1.34–29.37; *p* = 0.009), but there were no significant differences in mortality risks among patients in groups 2 and 3 compared with those in group 4. Conclusions: This preliminary study suggested that the GNRI may be used as a screening tool to identify patients with malnutrition at a high risk of mortality among elderly patients with femoral fractures. A prospective study is needed to validate the suggestion.

## 1. Background

Elderly individuals are at the greatest risk of a fall accidents [1,2] and their consequences, such as fractures, immobility, and sometimes even death [3,4,5,6,7]. In addition to pain, a femoral fracture results in a curtailment of physical activity, increased dependence, and rapid worsening of the health status. In addition, older people with femoral fractures are often malnourished at the time of fracture, and subsequently have poor food intakes. Malnutrition is frequently underdiagnosed in geriatric patients [8] and is considered to be a contributing factor for worse outcomes during hospitalization [9,10,11] or after fracture surgery [12,13]. Therefore, a screening tool to identify patients with a high risk of malnutrition is important while caring for elderly patients with femoral fractures. Over 70 tools have been proposed for assessing nutritional status, 21 of which were designed for use in elderly patients [14]. However, no tool is currently considered the gold standard for nutritional assessment [15]. Tools such as the Subjective Global Assessment, Nutritional Risk Screening (NRS), and Mini Nutritional Assessment (MNA) showed a good correlation with the overall picture of malnutrition in geriatric patients [16]. A comparison of the Mini Nutrition Assessment Short Form (MNA-SF), the Malnutrition Universal Screening Tool, and the NRS-2002 in 215 hip fracture-operated elderly patients revealed these three screening tools to be adequate for assessing malnutrition, but only the MNA-SF could additionally predict readmissions and mortality [17]. In a study of 437 admitted patients with a proximal femoral fracture, a comparison of the MNA-SF and the Short Nutritional Assessment Questionnaire (SNAQ), a questionnaire consisting of three questions concerning weight loss, appetite, and the use of dietary supplements according to the guidelines for elderly patients with femoral fractures [18], revealed no benefits of the SNAQ over the MNA-SF as a screening tool for malnutrition [19]. However, all of the above questionnaires have one prominent disadvantage, that is, it is difficult to complete them when the elderly patients have difficulties in communication, such as in the case of those who are unconscious or under intubation. Furthermore, some important information may not be recollected by the patients [20]. In a review of 44 studies involving 26,281 patients, the prevalence of malnutrition was approximately 45.7% when different criteria such as body mass index (BMI), weight loss, or albumin concentration were considered; however, it was approximately 18.7% using the MNA [21]. Thus, researchers may prefer to use the data of a physical measurement or a biochemical profile than a questionnaire to serve as a screening tool for nutritional assessment. The Nutritional Risk Index (NRI), calculated based on the level of albumin, current body weight, and usual body weight, was proposed for the evaluation of malnutrition status [22]. However, the score is often difficult to obtain because half of the elderly patients cannot recollect their own usual body weight [20]. An updated form of the NRI, the geriatric nutritional risk index (GNRI), was proposed by Bouillanne et al. to evaluate 6-month midterm nutritional outcomes of hospitalized elderly patients in a rehabilitation unit [23]. By replacing the usual body weight in the formula by the ideal body weight [23], the GNRI acts as a simple screening tool to assess the nutrition-related risk of morbidity and mortality in hospitalized elderly patients [24,25]. The GNRI has been validated to have a strong correlation with the mid-upper arm muscle circumference, arm muscle area, and handgrip strength of hospitalized patients [26], preoperative sarcopenia status of cancer patients [27], and MNA score [28]. The GNRI was reported to be a strong prognostic factor to assess the outcomes of various clinical conditions, including chronic kidney disease [29], heart failure [30], chronic obstructive pulmonary disease [31], chronic hemodialysis [32], and certain malignancies [33,34]. Serum albumin level has been recognized as a strong indicator of patients’ nutritional status. A low serum albumin level and total lymphocyte count at admission were significant predictors of one-year mortality in 174 elderly patients with intertrochanteric fractures [35]. A multiple logistic regression analysis in 127 patients who underwent surgery for femoral neck or trochanteric fractures revealed that the serum albumin level (OR = 5.9, *p* = 0.0004) and BMI (OR = 1.2, *p* = 0.0192) were significantly associated with mortality [36]. In a review of 19 studies involving 34,363 adults aged 74–85 years who underwent hip fracture surgery, the evaluation of predictive value of serum albumin level, total lymphocyte count at admission, and the MNA on patients’ nutritional status demonstrated that a low serum albumin level is a sole indicator of increased risks of postoperative complications, in-hospital mortality, and total mortality [37]. However, serum albumin levels can be influenced by the hydration status, inflammatory processes, and impairments of hepatic or renal function [28]. The GNRI is a simple tool based on two main components: the serum albumin level and the ratio of present body weight to ideal body weight. In addition to the albumin level, a second parameter is included in the GNRI to increase its predictive value. It was shown in a 3 year observational study conducted in institutionalized elderly that the GNRI had a higher prognostic value for mortality than albumin alone [38]. The ratio of present body weight to ideal body weight, the second parameter of the GNRI, may reflect the long-term nutritional condition of patients. Notably, pneumonia (37%) followed by acute coronary syndrome (31%) and sepsis (14%) are the most common causes of mortality in patients with hip fracture [39]. Death might be a consequence of nutrition-related complications [40,41]. Therefore, this study aimed to investigate whether the GNRI has a prognostic value that links the nutritional status and mortality outcomes in elderly patients who have previously undergone trauma with femoral fractures.

## 2. Methods

### 2.1. Ethics Statement

The study was approved before its implementation by the Institutional Review Board (IRB) of Chang Gung Memorial Hospital (approval number 202001446B0). Informed consent was waived according to the regulations of the IRB because the study was based on a retrospective analysis of registered data.

### 2.2. Data Extraction

**Figure 1:**
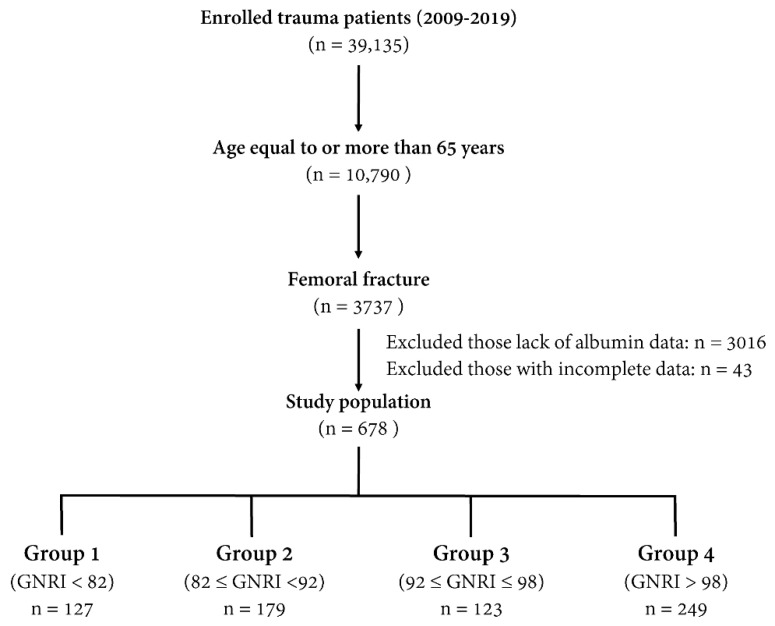
A flow chart illustrating the inclusion of elderly patients who had undergone trauma with femoral fractures, with the allocation of these patients into four nutritional risk groups.

Of the 39,135 enrolled patients with trauma-related injuries hospitalized for treatment between January 1, 2009 and December 31, 2019, 10,790 were elderly patients aged ≥65 years. Among these elderly patients, 3737 patients sustained a trauma femoral fracture. All types of femoral fractures due to the trauma injury were included in this study. After excluding the patients who had no recorded data of albumin levels (*n* = 3016) and who had incomplete data of body weight or pre-existing comorbidities (*n* = 43), 678 elderly patients with femoral fractures were enrolled in the study (Figure 1). The study population was categorized into four nutritional risk groups: a major-risk group (GNRI < 82; group 1, *n* = 127), moderate-risk group (GNRI 82 to <92; group 2, *n* = 179), low-risk group (GNRI 92–98; group 3, *n* = 123), and no-risk group (GNRI > 98; group 4, *n* = 249) according to the original description provided by Bouillanne et al. [23]. The following medical information of these patients was extracted from the Trauma Registry System of the hospital [42,43,44]: sex, age, BMI, albumin level on admission, preexisting comorbidities (diabetes mellitus (DM), hypertension (HTN), coronary artery disease, congestive heart failure, cerebral vascular accident (CVA), and end-stage renal disease), Glasgow coma scale (GCS) score, injury severity score (ISS), and in-hospital mortality. ISS was obtained from the sum of the squares of the three highest abbreviated injury scale scores of different body regions and represented the injury severity of the patients [45,46,47]. The GNRI was calculated as follows: (1.489 × albumin (g/dl) + 41.7 × (body weight/ideal body weight)) originally proposed by Bouillanne et al. [23].

### 2.3. Statistical Analysis

The data set for continuous variables was analyzed by the Kolmogorov–Smirnov test for distribution of normalization. Analysis of variance and the Mann–Whitney U-test were used to analyze normally and non-normally distributed continuous data, respectively, with Bonferroni post hoc correction. The results are expressed as mean ± standard deviation or medians and interquartile ranges (IQR, Q1–Q3). Categorical data were compared using two-sided Fisher’s exact or Pearson χ^2^ tests with the presentation of odds ratios (ORs) and 95% confidence intervals (CIs). To minimize the confounding effects of sex, age, preexisting comorbidities, and injury severity of patients on outcome measurements, a logistic regression model was used to calculate the propensity scores with the above covariates and a 1:1 propensity score-matched patient cohort against the no-risk group (group 4) were created using the NCSS 10 software (NCSS statistical software, Kaysville, UT, USA) with the greedy method and a 0.2-caliper width. These matched patient cohorts were used to assess the impact of patients being in different nutritional risk groups on mortality outcomes in the no-risk group. In this study, the in-hospital mortality of patients was defined as the primary outcome and all statistical analyses were performed using Windows version 23.0 for SPSS (IBM Inc., Chicago, IL, USA). *p* values < 0.05 were considered statistically significant.

## 3. Results

### 3.1. Patient and Injury Characteristics

A comparison of the groups with different risks for malnutrition in the study population showed that there was a significantly higher percentage of male patients in group 1 than in group 4 (Table 1). The patients in groups 1–3 were significantly older and presented a significantly lower BMI and level of albumin than the patients in group 4. There were significant intergroup differences in the prevalence of preexisting comorbidities of DM, HTN, and CVA. Additionally, there were significant intergroup differences in the GCS scores (*p* = 0.016), with a significantly lower GCS score in group 2 than in group 4. When stratified by GCS scores (3–8, 9–12, or 13–15), significantly more patients had GCS scores of 9–12 in group 1 and group 2, but fewer patients had GCS scores of 13–15 in group 2 than in group 4. There was no significant difference in the ISS among these groups of patients (*p* = 0.705) regardless of ISS stratification (1–15, 16–24, and ≥25). Compared to the patients in group 4, a significantly higher mortality rate was found in those in group 1 (17.3% vs. 3.6% for group 1 vs. group 4 patients, respectively, *p* < 0.001), but not in those in group 2 (6.7%) or group 3 (2.4%).

### 3.2. Comparison of Propensity Score-Matched Patient Cohorts

To minimize the confounding effects of sex, age, preexisting comorbidities, and injury severity of patients on outcome measurements, a 1:1 propensity score-matched patient cohort was created separately for the patients in groups 1–3 against those in group 4 (Supplemental Tables S1–S3). The selected pairs of propensity score-matched patient populations were those who did not present significant differences in sex, age, comorbidities, and ISS (Supplemental Tables S1–S3). As shown in Table 2, among the selected 75 pairs of patients, the patients in group 1 had significantly higher odds of mortality than those in group 4 (OR, 6.3; 95% CI, 1.34–29.37; *p* = 0.009). The patients in group 2 and group 3 did not have significantly different odds of mortality than those in group 4 among the selected 95 and 88 pairs of patients, respectively, in pairs of matched patient populations.

## 4. Discussion

The study results demonstrated that the GNRI helps classify malnutrition risk in elderly patients who had undergone trauma with femoral fractures. In elderly trauma patients with femoral fractures, the patients with GNRI < 82 presented with a significant 6.3-fold adjusted mortality risk compared with the patients in the no-risk group whose GNRI > 98. The cutoff value was chosen according to the recommendations of Bouillanne et al., who determined four cutoff values for GNRI (GNRI < 82, GNRI 82 to <92, GNRI 92–98, and GNRI > 98) to indicate the risk of malnutrition [23]. However, some different GNRI cutoff values have been reported for various diseases or clinical situations [48,49,50]. In patients undergoing hemodialysis, a GNRI < 100 was significantly associated with mortality outcomes (hazard ratio 3.691; 95% CI, 1.75–7.78; *p* = 0.001) [51]. In patients undergoing pancreaticoduodenectomy, a GNRI < 94 was an independent predictor of surgical site infection (relative risk 1.73; 95% CI, 1.23–2.43; *p* < 0.001) [52]. For elderly patients with sepsis in the acute-care setting, a GNRI < 87 has been proposed as the optimal cutoff indicating the requirement of nutritional support [53].

This study has some limitations and thus the results may be regarded as preliminary and require further validation. First, the retrospective design of this study may lead to a selection bias, especially considering that a large proportion of patients with femoral fractures did not have serum albumin data at admission. However, this also reflects that the assessment of the nutritional status is often neglected even for the patients with femoral fractures. Second, the trauma registry data only recorded in-hospital mortality but not data of 30 days or beyond; thus, the results may not reflect the full scope of the mortality associated with femoral fractures in the elderly. In addition, the patients declared dead on arrival in the emergency room were not included in the database, which may have led to a selection bias in the outcome measurement. Third, this study did not investigate interventions such as resuscitation, immobilization, and surgery conducted on patients; thus, the outcome measurements may be biased. Under these circumstances, we can only assume that the outcomes of the treatments were uniform across the study population. Fourth, a selection bias may occur because most patients with femoral fractures in the database did not have records of albumin levels and thus were excluded from the study; however, these data also reflected that the nutrition status was generally neglected by the orthopedists and trauma surgeons. Finally, the population included in this study was limited to that of a single urban trauma center, and the results may not be applicable to a wider population.

## 5. Conclusions

This preliminary report suggests that the GNRI may be used as a screening tool to identify patients with malnutrition at a high risk of mortality among elderly patients with femoral fractures. A prospective study is needed to validate the finding.

## Figures and Tables

**Table 1:** Patient and injury characteristics of the groups of patients with different risks of malnutrition.

Variables	Group 1	Group 2	Group 3	Group 4	*p*
Range of GNRI	GNRI < 82	82 ≤ GNRI < 92	92 ≤ GNRI ≤ 98	GNRI > 98	
Number of patients	*n* = 127	*n* = 179	*n* = 123	*n* = 249	
Gender					0.033
Male, *n* (%)	53 (41.7) *	67 (37.4)	43 (35.0)	69 (27.7)	
Female, *n* (%)	74 (58.3) *	112 (62.6)	80 (65.0)	180 (72.3)	
Age (years)	81.8 ± 7.3 *	81.3 ± 7.5 *	78.9 ± 6.9	77.4 ± 7.5	<0.001
BMI	18.8 ± 2.9 *	21.9 ± 2.8 *	23.4 ± 3.0 *	26.6 ± 3.9	<0.001
Albumin level (g/dL)	2.6 ± 0.5 *	3.0 ± 0.5 *	3.3 ± 0.5 *	3.8 ± 0.5	<0.001
Comorbidities					
DM, *n* (%)	28 (22.0) *	50 (27.9) *	46 (37.4)	110 (44.2)	<0.001
HTN, *n* (%)	67 (52.8) *	113 (63.1)	90 (73.2)	178 (71.5)	0.001
CAD, *n* (%)	16 (12.6)	18 (10.1)	20 (16.3)	37 (14.9)	0.374
CHF, *n* (%)	6(4.7)	8 (4.5)	6 (4.9)	7 (2.8)	0.695
CVA, *n* (%)	12 (9.4)	20 (11.2)	29 (23.6) *	27 (10.8)	0.002
ESRD, *n* (%)	2 (1.6)	15 (8.4)	5 (4.1)	14 (5.6)	0.063
GCS, median (IQR)	15 (15–15)	15 (15–15) *	15 (15–15)	15 (15–15)	0.016
3–8	1 (0.8)	6 (3.4)	1 (0.8)	4 (1.6)	0.265
9–12	8 (6.3) *	11 (6.1) *	3 (2.4)	1 (0.4)	0.002
13–15	118 (92.9)	162 (90.5) *	119 (96.7)	244 (98)	0.003
ISS, median (IQR)	9 (9–9)	9 (9–9)	9 (9–9)	9 (9–9)	0.705
1–15	121 (95.3)	165 (92.2)	114 (92.7)	230 (92.4)	0.718
16–24	1 (0.8)	4 (2.2)	3 (2.4)	9 (3.6)	0.417
≥25	5 (3.9)	10 (5.6)	6 (4.9)	10 (4.0)	0.863
Mortality, *n* (%)	22 (17.3) *	12 (6.7)	3 (2.4)	9 (3.6)	<0.001

BMI = Body mass index; CAD = coronary artery disease; CHF = congestive heart failure; CI = confidence interval; CVA = cerebral vascular accident; DM = diabetes mellitus; ESRD = end-stage renal disease; GCS = Glasgow Coma Scale; GNRI = geriatric nutritional risk index; HTN = hypertension; IQR = interquartile range; ISS = injury severity score; OR = odds ratio. * indicates a *p* < 0.05 in comparison with Group 4.

**Table 2:** Comparison of the mortality outcomes in the propensity-score matched cohorts of the elderly patients with femoral fractures in group 1–3 vs. group 4 patients.

Groups of Comparison	Numbers of Pairs of Patients *n*	Mortality of Compared Group *n* (%)		Mortality of Group 4 *n* (%)		OR (95% CI)		*p*
Group 1 vs. Group 4	**75**	**11**	(14.7)	2	(2.7)	6.3	(1.34–29.37)	0.009
Group 2 vs. Group 4	95	7	(7.4)	2	(2.1)	3.7	(0.75–18.29)	0.088
Group 3 vs. Group 4	88	2	(2.3)	5	(5.7)	0.4	(0.07–2.05)	0.247

## Supplementary Materials

The following are available online at https://www.mdpi.com/1660-4601/17/23/8920/s1, Table S1: Comparison of the mortality outcomes in the propensity-score matched cohorts of the elderly patients with femoral fractures in group 1 vs. group 4 patients; Table S2: Comparison of the mortality outcomes in the propensity-score matched cohorts of the elderly patients with femoral fractures in group 2 vs. group 4 patients; Table S3: Comparison of the mortality outcomes in the propensity-score matched cohorts of the elderly patients with femoral fractures in group 3 vs. group 4 patients.
