# Supplementary material for: Geriatric Nutritional Risk Index as a Screening Tool to Identify Patients with Malnutrition at a High Risk of In-Hospital Mortality among Elderly Patients with Femoral Fractures—A Retrospective Study in a Level I Trauma Center
Source: Int J Environ Res Public Health. 2020 Nov 30;17(23):8920. doi: 10.3390/ijerph17238920 (PMC7729938; doi:10.3390/ijerph17238920)
Supplement: Supplementary file 1 [file ijerph-17-08920-s001.pdf]

Table S1. Comparison of the mortality outcomes in the propensity-score matched cohorts of the elderly patients with femoral fractures in group 1 vs. group 4 patients.

Matched cohorts by propensity score								
**Variables**	**Group 1** *n* = 75		**Group 4** *n* = 75		**OR (95% CI)**		***p***	**Standardized Difference**
Male, *n* (%)	24	(32.0)	24	(32.0)	1.0	(0.50–1.99)	1.000	0.00%
Age, years	80.7	±6.6	80.6	±6.8		-	0.971	0.60%
Comorbidities								
CVA, *n* (%)	4	(5.3)	4	(5.3)	1.0	(0.24–4.16)	1.000	0.00%
HTN, *n* (%)	45	(60.0)	45	(60.0)	1.0	(0.52–1.92)	1.000	0.00%
CAD, *n* (%)	5	(6.7)	5	(6.7)	1.0	(0.28–3.61)	1.000	0.00%
CHF, *n* (%)	0	(0.0)	0	(0.0)		-	-	-
DM, *n* (%)	13	(17.3)	13	(17.3)	1.0	(0.43–2.33)	1.000	0.00%
ESRD, *n* (%)	0	(0.0)	0	(0.0)		-	-	-
GCS, median (IQR)	15	(15–15)	15	(15–15)		-	0.940	1.23%
ISS, median (IQR)	9	(9–9)	9	(9–9)		-	1.000	0.00%

BMI = Body mass index; CAD = coronary artery disease; CHF = congestive heart failure; CI = confidence interval; CVA = cerebral vascular accident; DM = diabetes mellitus; ESRD = end-stage renal disease; HTN = hypertension; IQR = interquartile range; ISS = injury severity score; OR = odds ratio.

Table S2. Comparison of the mortality outcomes in the propensity-score matched cohorts of the elderly patients with femoral fractures in group 2 vs. group 4 patients.

Matched cohorts by propensity score								
**Variables**	**Group 2** *n* = 95		**Group 4** *n* = 95		**OR (95% CI)**		***p***	**Standardized Difference**
Male, *n* (%)	25	(26.3)	25	(26.3)	1.0	(0.52–1.91)	1.000	0.00%
Age, years	81.1	±6.9	80.8	±7.1		-	0.731	4.99%
Comorbidities								
CVA, *n* (%)	5	(5.3)	5	(5.3)	1.0	(0.28–3.57)	1.000	0.00%
HTN, *n* (%)	71	(74.7)	71	(74.7)	1.0	(0.52–1.92)	1.000	0.00%
CAD, *n* (%)	3	(3.2)	3	(3.2)	1.0	(0.20–5.08)	1.000	0.00%
CHF, *n* (%)	0	(0.0)	0	(0.0)		-	-	-
DM, *n* (%)	25	(26.3)	25	(26.3)	1.0	(0.52–1.91)	1.000	0.00%
ESRD, *n* (%)	2	(2.1)	2	(2.1)	1.0	(0.14–7.25)	1.000	0.00%
GCS, median (IQR)	15	(15–15)	15	(15–15)		-	0.578	8.08%
ISS, median (IQR)	9	(9–9)	9	(9–9)		-	0.847	-2.80%

BMI = Body mass index; CAD = coronary artery disease; CHF = congestive heart failure; CI = confidence interval; CVA = cerebral vascular accident; DM = diabetes mellitus; ESRD = end-stage renal disease; HTN = hypertension; IQR = interquartile range; ISS = injury severity score; OR = odds ratio.

Table 3. Comparison of the mortality outcomes in the propensity-score matched cohorts of the elderly patients with femoral fractures in group 3 vs. group 4 patients.

Matched cohorts by propensity score								
**Variables**	**Group 3** *n* = 88		**Group 4** *n* = 88		**OR (95% CI)**		***p***	**Standardized Difference**
Male, *n* (%)	26	(29.5)	26	(29.5)	1.0	(0.52–1.91)	1.000	0.00%
Age, years	78.4	±6.7	78.3	±6.6		-	0.928	1.37%
Comorbidities								
CVA, *n* (%)	10	(11.4)	10	(11.4)	1.0	(0.39–2.54)	1.000	0.00%
HTN, *n* (%)	72	(81.8)	72	(81.8)	1.0	(0.47–2.15)	1.000	0.00%
CAD, *n* (%)	13	(14.8)	13	(14.8)	1.0	(0.44–2.30)	1.000	0.00%
CHF, *n* (%)	1	(1.1)	1	(1.1)	1.0	(0.06–16.24)	1.000	0.00%
DM, *n* (%)	33	(37.5)	33	(37.5)	1.0	(0.54–1.84)	1.000	0.00%
ESRD, *n* (%)	2	(2.3)	2	(2.3)	1.0	(0.14–7.26)	1.000	0.00%
GCS, median (IQR)	15	(15–15)	15	(15–15)		-	0.566	8.68%

ISS, median (IQR)	9	(9–9)	9	(9–9)	-	0.428	11.99%

BMI = Body mass index; CAD = coronary artery disease; CHF = congestive heart failure; CI = confidence interval; CVA = cerebral vascular accident; DM = diabetes mellitus; ESRD = end-stage renal disease; HTN = hypertension; IQR = interquartile range; ISS = injury severity score; OR = odds ratio.
